# Evaluating the feeding preferences of West Nile virus mosquito vectors using bird-baited traps

**DOI:** 10.1186/s13071-016-1744-6

**Published:** 2016-08-31

**Authors:** Isis Victoriano Llopis, Laura Tomassone, Elena Grego, Emmanuel Serrano, Andrea Mosca, Gabriella Vaschetti, Daniela Andrade, Luca Rossi

**Affiliations:** 1 Dipartimento di Scienze Veterinarie, University of Turin, Turin, Italy; 2 CESAM, Departamento de Biologia, Universidade de Aveiro, Aveiro, Portugal; 3 Servei d’ Ecopatologia de Fauna Salvatge, Departamento de Medicina y Cirugia Animal, Universidad Autónoma de Barcelona (UAB), Bellaterra, Barcelona, Spain; 4 Istituto per le Piante da Legno e l’Ambiente, IPLA spa, Turin, Italy; 5 Associazione Centro Cicogne e Anatidi di Racconigi, Racconigi, Italy

**Keywords:** *Aedes* spp., *Anopheles* spp., *Culex* spp., *Ochlerotatus* spp., Italy, Mosquito attraction, WNV, Raptors, Vector-borne diseases

## Abstract

**Background:**

The total contact rates (TCRs) between mosquito vectors and their potential hosts have a serious impact on disease transmission dynamics. *Culex pipiens* (*sensu stricto*) (*s.s*.) is considered the main vector of the West Nile Virus (WNV) in Europe and birds are the reservoir hosts. The results of our previous study showed that WNV seroreactors are significantly more prevalent among raptors compared to a range of other wild avian groups. The current study aims to assess the role of bird type (raptor *vs* others) and bird size on mosquito feeding preferences in a free-choice experiment using bird-baited traps.

**Methods:**

From July to September 2014, a battery of six bird-baited traps was operated in twelve mosquito capture sessions. Eight bird species, belonging to five different orders, including raptors, were used. After each session, the trapped mosquitoes were collected and identified using standard keys. Two sets of independent generalized linear mixed models (GLMM) were used to assess mosquito vector feeding preferences (MFp) among different bird species and types.

**Results:**

A total of 304 mosquitoes belonging to seven taxa were collected, *C. pipiens* being by far the most abundant (84.2 % of the total mosquito catch). Most *C. pipiens* were engorged (83.59 %). The selected model showed that 25.6 % of the observed variability of MFp is explained by the interaction between bird size and bird type, with *C. pipiens* preferring to feed on large birds, especially raptors. The proportion of engorged mosquitoes was 1.9-fold higher in large (22.88 %; range 0–42 %) than in medium-sized raptors (11.71 %; range 0–33 %), and was nearly the same in medium-sized (9.08 %; range 0–26 %) and large (8.5 %; 6–24 %) non-raptor species.

**Conclusion:**

*Culex pipiens* showed an obvious preference for large raptors, which concurs with the higher seroprevalence to WNV in our previous study. The appreciable feeding by *C. pipiens* on large raptors makes them useful alternative sentinels to poultry for WNV surveillance. Thus, wildlife parks and rehabilitation centers can contribute to surveillance efforts to a greater extent.

## Background

West Nile Virus (WNV) is now one of the most common causes of epidemic viral encephalitis and has the most widespread geographical distribution of all mosquito-borne flaviviruses [1]. This virus belongs to the Japanese encephalitis virus antigenic complex of the family Flaviviridae, the genus *Flavivirus*. Several WNV outbreaks have been reported over the past few decades in European countries, including Italy, where the virus is endemic in the northeastern regions [2]. The virus is maintained in the environment through a bird-mosquito life-cycle and *Culex pipiens* (*sensu stricto*) (*s.s*.) is considered the main vector in Europe [3]. Humans and horses are incidental hosts. Although most of the infections in these species are asymptomatic, some cases develop fever or fatal encephalitis [4]. Different migratory and resident avian species disseminate and amplify WNV, and the degree, duration of viremia and susceptibility to infection vary by species [5, 6]. Diurnal and nocturnal birds of prey were found to be frequently infected by WNV during outbreaks in North America and Europe [7–11].

Several studies on mosquito host preference have been performed to investigate the WNV transmission cycle, in order to determine which avian species are involved in WNV epidemics [12, 13]. Few studies, however, have focused on the host selection of mosquitoes in wild birds using bird-baited traps, which requires a large collection of birds and repeated trials. Most field studies on mosquito blood-feeding patterns on birds have been carried out in passeriform species, and the presence of raptors in these studies is uncommon [14].

The assessment of total contact rates (TCRs) between mosquito vectors and their potential hosts is key, not only to understanding vector-borne disease dynamics, but also to optimize efforts in pathogen monitoring in the wild [15]. Several studies have shown that mosquitoes rarely feed at random [12, 16, 17], and that olfaction of specific odorants and skin emanations drives host selection [18]. Although host choice in mosquito vectors is often biased in favour of larger animals, because of the higher quantities of exhaled CO_2_ [19], other factors such as body heat and defensive behaviors can play a role in host selection [20]. On the other hand, mosquito feeding behaviors are also driven by the diversity and abundance of blood sources, by mosquito abundance, and their interaction [19, 20].

Direct assessment of mosquito feeding patterns has been studied by choice assays, in which mosquitoes are exposed to different olfactory sources in laboratory conditions (e.g. live hosts or host-derived odor samples). Even though these controlled assays involve complex procedures (e.g. olfactometers, indoor observational rooms, choice chambers or wind tunnels [21]), they only take into account part of the host stimuli, neglecting other environmental factors linked to mosquito biological activity [20].

In field conditions, feeding preferences may be assessed by the analysis of blood meals of field-collected mosquitoes [12]. This approach, however, is often biased in favour of the most abundant host species, and the challenging assessment of host availability is required to understand the absolute feeding preferences of vectors [20].

Animal-baited traps (i.e. traps covered with mosquito nets that allow the entry of host-seeking mosquitoes), have also been widely used to assess feeding preferences in field conditions. Although this method allows mosquitoes to select among several hosts, it is also a subject to some limitations, e.g. nets are often raised above the ground to allow their entry, mosquitoes may escape, and engorged individuals may not necessarily have fed from the host present in the baited-trap [22, 23]. However, animal-baited traps can be considered unbiased because of the possible encounter with more than one host-seeking mosquito and more than one potential blood source. In fact, mosquitoes are not only attracted by the CO_2_ exhaled by animals, but also by their odor and visual cues. This method allows the study of preferences for one individual among others of the same species, minimizing the potential bias due to natural intra-specific odor variations. Finally, animal-baited traps are a cheap and easy method of catching mosquitoes attracted to a variety of animals [23].

Recently, we conducted a seroprevalence study in 871 individuals of 87 different bird species in northwestern Italy. The results of this study showed that WNV seroreactors were significantly more prevalent amongst raptors (orders Falconiformes, Strigiformes and Accipitriformes) compared to other avian groups [24]. To investigate the hypothesis that mosquitoes in the area preferentially feed on raptor birds, thus justifying higher seropositivity to WNV in response to higher TCRs, we planned an experiment allowing free-choice feeding of wild mosquitoes on raptors and other avian species belonging to five different orders (Passeriformes, Strigiformes, Columbiformes, Falconiformes and Anseriformes). Taking advantage of this controlled experiment in semi-natural conditions, we studied the role of bird type (raptor species *vs* others) and bird size in the attraction and feeding of *Culex pipens* and other potential WNV mosquito vectors. The role of bird traits in the richness of mosquito species attracted and their abundance was also evaluated. The operative and ultimate goal of the study was exploring if raptors, which are frequently admitted patients at rehabilitation centers in Europe, and are often cared for in these facilities for years or lifelong if unfit for release in the wild [25–27], may represent appropriate wildlife to consider by public health agencies for enhanced surveillance of WNV and other zoonotic flaviviruses.

## Methods

### Experimental assessment

The experiment was carried out at a wildlife recovery center situated in “Centro Cicogne e Anatidi” in Racconigi, Piedmont region, northwestern Italy (44.7779N, 7.6691E). This location was selected because of the high abundance of ornithophilic mosquitoes and because it allows a large collection of wild captive birds in an open mosquito-accessible environment.

### Bird-baited traps

From 30 July 2014 to 10 September 2014, six bird-baited traps were set contemporaneously in twelve sessions, from 19:00 to 8:00. Three of these were baited with different raptor species and the remaining three were baited with non-raptor species. A total of eight bird species were used in the experiment: the peregrine falcon (*Falco peregrinus*), the common kestrel (*Falco tinnunculus*), the little owl (*Athene noctua*), the mallard (*Anas platyrhynchos*), the European blackbird (*Turdus merula*), the song thrush (*Turdus philomelos*), the diamond dove (*Geopelia cuneata*) and the Eurasian collared dove (*Streptopelia decaocto*). Individuals of these species are frequently among long-term patients of the center where we operated. Very small-sized birds [below 40 g body weight (b.w.)] were not used since they are rarely hospitalized in this and other rescue centers, but as nestlings.

For the experiments, birds were transferred to the outdoor cages of the recovery center. Cages were situated in a garden near ponds for waterfowl in an open environment. These cages measured 2 × 1.3 × 2 m and were similar to the original cages where birds had been placed, in order to minimize bird stress. They were placed adjacent to each other and the walls were made of cement. Doors, constructed of grating, were on the front of the cage and were covered with mosquito nets, raised 10 cm from the ground to allow the entry of host-seeking mosquitoes. To reduce the possibility of mosquito exit, nets only had a single entrance and birds were not enclosed within an inner protective net. To minimize the potential bias due to cage positions and individual characteristics of the animals, bird placement within the six different cages was randomized, and when possible, different individuals of the same species were used in each trial. After each trial, the trapped mosquitoes were mechanically aspirated using battery-operated aspirators and the birds were returned to their holding cages. We used only birds with minor injuries and in good general condition to minimize the risk of harm due to the stress of handling. All mosquitoes captured during the study were stored frozen at -80 °C for later taxonomic identification using standard keys [28, 29].

### Statistical analysis

The prevalence of mosquito species and engorged specimens from bird-baited traps was calculated, with 95 % exact binomial confidence intervals (95 % CI). Bird species were divided into groups according to their size (small birds: < 100 g; medium-sized birds: 100–250 g; and large birds: > 500 g) and to bird type, i.e. raptors (peregrine falcon, common kestrel and little owl) and other species (European blackbird, song thrush, Eurasian collared dove, mallard and diamond dove). To assess WNV mosquito vector feeding preferences (MFp) in different bird species, we fitted two sets of independent generalized linear mixed models (GLMM) in which the observed variability of MFp or mosquito species richness (Mrich), as response variables, were explained by the single fixed effects of bird type (i.e. raptors and other species), bird size (Bs), and their two-way interactions. Mosquito populations in temperate ecosystems are temporally variable, hence we first checked for the potential confounding effects of date of sampling on MFp by a linear model. Our results showed a lack of temporal patterns (*β* = -0.201, *SE* = 0.06, *t*-value = -0.98, *P* = 0.32) on MFp and thus the factor was not included in our model selection.

The random term was the individual bird nested in the baited trap; in other words, a repeated measures fixed-block design was used [30]. The best random structure (intercept, slope or both) was selected following the procedure described by Zuur et al. [31].

No small raptors were present in our sample and thus, only medium-sized and large species were retained in our statistical modelling. Since *Culex pipiens* was by far the most common mosquito species, MFp was defined as the percentage of engorged *C. pipiens* (MFp = 100 × engorged *C. pipiens*/total *C. pipiens*) by trap. Mrich, however, was calculated as the number of different mosquito species by trapping session.

Models for MFp were fitted using a normal distribution and the identity link function. The models for Mrich were fitted with a log-link and Poisson errors after checking for the absence of overdispersion in the data (residual deviance greater than the residual degrees of freedom; [31]). For all statistical models, we performed a model selection procedure based on the information-theoretic approach and Akaike’s Information Criterion corrected for small sample sizes (AICc) [32]. In short, competing models are ranked in relation to the difference between their Akaike scores and the score of the best model (i), which has the lowest AICc. Models with i < 2 units have substantial support for explaining the observed variability in the variables of interest. Subsequently, we estimated the Akaike weight (wi), defined as the relative probability that a given model is the best model of those being compared. In addition, pseudo-R^2^ values for the fixed (marginal variance) and both for the fixed and random factors (conditional variance), were also calculated for the best GLMM as an overall measure of goodness-of-fit following the procedure described by Nakagawa & Schielzeth [33]. Finally, once the best model was selected, we checked the lack of the residual patterns. Models were carried out using the “*nlme*” [34], “*lme4*” [35] and “*MuMln*” [36] packages of the R statistical software version 3.2.3 [37].

## Results

A total of 304 mosquitoes belonging to seven species were collected during the bird-baited traps experiment (Table 1). *Culex pipiens* was by far the most common species, representing 84.2 % of the total mosquito catch followed by *Anopheles maculipennis* (*sensu lato*) (*s.l*.) (3.3 %), *Aedes albopictus* (3.3 %) and *Aedes vexans* (2.6 %). The least abundant species were *Culex territans* (1.9 %), *Culex modestus* (1.6 %) and *Ochlerotatus geniculatus* (1.3 %). The majority of *C. pipiens* collected were engorged with blood (83.59 %). Moreover, *A. albopictus* and *C. modestus* showed a high percentage of engorged specimens (60.0 %), followed by *A. maculipennis* (*s.l*.) (50.0 %). In contrast, most of the collected specimens of *O. geniculatus* and *A. vexans* were unfed since the percentages of engorged specimens were 25 % and 12.5 %, respectively. All *C. territans* were unengorged (Table 1). Five mosquito specimens were damaged and it was impossible to identify them. The number of mosquitoes captured per bird species is shown in Table 2. The range of percentage of *C. pipiens* feeding preferences and the percentage of engorged mosquitoes by bird species are summarized in Table 3.

**Table 1:** Mosquito species, % of total of mosquito captures, % of engorged mosquitoes and feeding preference behavior

Mosquito species	% of total mosquito captures (95 % CI)	% of engorged mosquitoes (95 % IC)	Feeding preference
*Culex pipiens*	84.2 (79.6–88.1)	83.59 (78.5–87.9)	Birds and humans [58]
*Aedes albopictus*	3.3 (1.6–5.9)	60.0 (26.2–87.8)	Humans [59]
*Anopheles maculipennis* (*s.l*.)	3.3 (1.6–5.9)	50.0 (18.7–81.3)	Mammals, rarely on humans and birds [22]
*Aedes vexans*	2.6 (1.1–5.1)	12.5 (0.3–52.6)	Mammals [60]
*Culex territans*	1.9 (0.7–4.2)	unengorged	Amphibians [61]
*Culex modestus*	1.6 (0.5–3.8)	60.0 (14.6–94.7)	Opportunistic [15, 22]
*Ochlerotatus geniculatus*	1.3 (0.3–3.3)	25.0 (0.6–80.6)	Humans and cattle [62]

**Table 2:** Number of mosquitoes attracted by each bird species

Bird species	Number of trials	*C. pipiens*	*Ae. albopictus*	*An. maculipennis* (*s.l*.)	*Ae. vexans*	*C. territans*	*C. modestus*	*O. geniculatus*	NI^a^
*Falco peregrinus*	12	77	2	2	3	1	2	2	1
*Falco tinnunculus*	12	40	4	1	1	2	0	1	2
*Turdus philomelos*	5	17	1	2	2	0	0	0	0
*Athene noctua*	12	43	1	1	2	1	0	0	0
*Turdus merula*	7	22	1	4	0	0	1	0	0
*Streptopelia decaocto*	11	25	0	0	0	2	0	1	2
*Anas platyrhynchos*	12	30	1	0	0	0	2	0	0
*Geopelia cuneata*	1	2	0	0	0	0	0	0	0
Total		256	10	10	8	6	5	4	5

^a^ NI, specimens impossible to identify

**Table 3:** Number of trials and number of different individuals for each bird species, range of % of attracted *C. pipiens* in each bird species (mosquito specimens captured on each bird/mosquito specimens captured on all birds in the same session), % of engorged mosquitoes and size of the birds: small (< 100 g), medium (100–250 g), large (> 500 g)

Bird species			No. of trials	No. of different birds	Range of % of attracted mosquitoes	% engorged mosquitoes (95 % IC)
Raptors	Medium	*Athene noctua*	12	5	0–39.29	93.02 (80.93–98.53)
	Medium	*Falco tinnunculus*	12	3	0–28.57	78.37 (61.78–90.17)
	Large	*Falco peregrinus*	12	4	23.53–42.86	88.15 (78.70–94.43)
Other	Small	*Turdus merula*	7	3	0–23.26	86.36 (65.08–97.09)
	Small	*Turdus philomelos*	5	2	0–23.53	88.23 (63.55–98.54)
	Small	*Geopelia cuneata*	1	1	10.53	100 (15.81–100)
	Medium	*Streptopelia decaocto*	11	11	0–66.67	79.16 (57.84–92.86)
	Large	*Anas platyrhynchos*	12	7	0–21.05	82.14 (63.10–93.93)

**Fig. 1:**
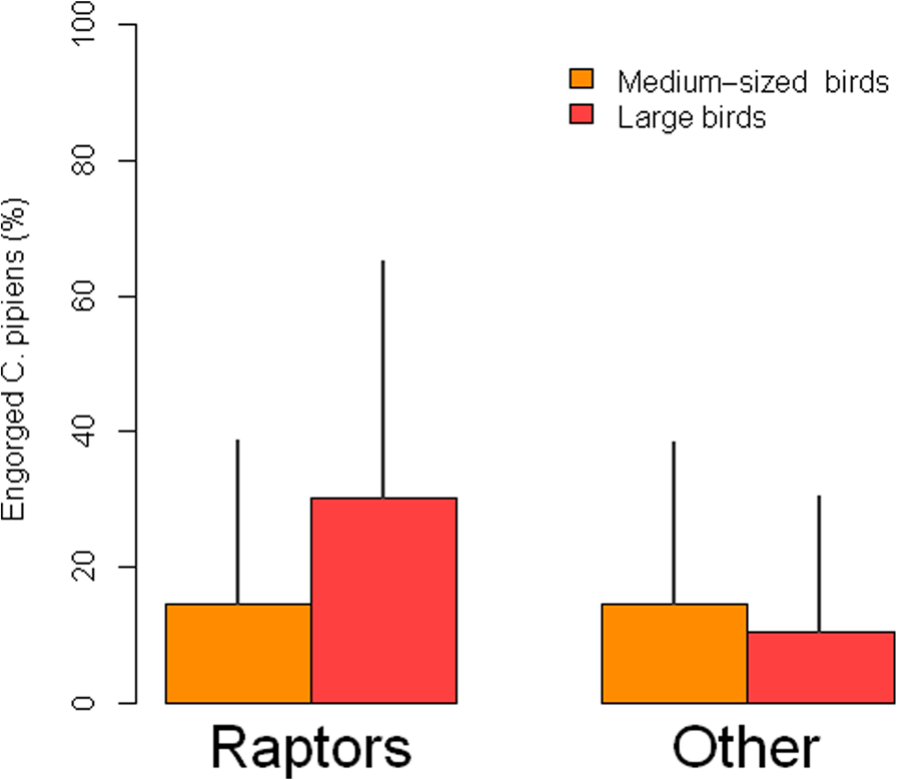
Bar plots representing differences of *Culex pipiens* feeding preferences (MFp) in raptor and non-raptor species. Two categories of size were considered: medium-sized (body weight 100–250 g) and large birds (body weight > 500 g). MFp was defined as the percentage of engorged *Culex pipiens* (MFp = 100 × engorged *C. pipiens*/total *C. pipiens*) by trap. Bars represent the mean MFp and whiskers the associated standard error of the mean

Our model selection showed that 25 % of the observed variability of MFp can be explained by the interaction between bird size and bird type (Wi _Bird type * Bird size_ = 0.96, *β* = -0.201, *SE* = 0.06, *t*-value = -3.02) (Table 4). The selected model indicates that *C. pipiens* prefer to feed on large birds, especially among raptors (Fig. 1). The proportion of engorged mosquitoes and trapped in cages containing each bird type is shown in Table 5. Finally, a very low proportion of the variance in the observed relationships between size, type and feeding patterns was due to the random terms (1.72e^-09^ for the cage and 1.21e^-09^ for the trial effects). As a result, both marginal and conditional pseudo-R^2^ resulted identical. Regarding Mrich, no model was able to explain the observed patterns in the number of mosquito species collected in our experiment.

**Table 4:** Model selection for assessing mosquito feeding preferences (MFp) in the two types of bird species (Bird type: raptors *vs* other species) with medium (body weight between 100 to 250 g) and large (body weight over 500 g) size (Bs)

Biological models	K	AICc	∆i	wi	Pseudo R^2^	
					Marginal	Conditional
Bird type * Bird size	7	−69.07	0	0.96	25.6	25.6
Bird size	5	−60.83	9.28	0.01	4.2	4.2
Bird type	5	−59.17	10.94	<0.01	6.9	6.9
Mo	4	−58.96	11.53	<0.01	0	0

K = number of parameters, AICc = Akaike Information Criterion corrected for small sample sizes, ∆i = difference of AICc with respect to the best model, wi = Akaike weight, Pseudo-R^2^ = percentage of observed variability in the response variable explained only by the fixed terms (Marginal) or by both fixed and random terms (Conditional). In bold, models with substantial support for being the best model. In the null model (Mo), all of the terms are excluded except the intercept

**Table 5:** Mean, minimum and maximum percentage of engorged mosquitoes in bird types (raptors *vs* other species) with different bird size: medium (body weight between 100 to 250 g) and large (body weight over 500 g)

Bird size	Raptors	Non-raptors	Total
Medium	11.71 (0–33)	9.08 (0–26)	10.39
Large	22.82 (0–42)	8.5 (6–24)	15.66
Total	17.26	8.79	

## Discussion

Mosquito feeding on raptor *vs* non-raptor birds was investigated in a free-choice experiment under semi-natural conditions. A major finding was that *C. pipiens*, one of the main vectors of arboviral zoonotic diseases [38], fed preferentially on raptors over other birds in the study. Several surveys conducted in North America revealed that raptors constitute a small percentage of total avian blood meals of mosquitoes collected in the field, varying from 0.3 to 3.4 %, as expected for low density hosts on top of the food chains [39–42]. However, a study of mosquito blood meal analysis at a raptor rehabilitation center in Alabama (USA), reported that more than 58 % of blood meals were from raptors, showing that the frequency of raptor blood meals is clearly influenced by their abundance in the avian community [19]. The results of our experiment suggest that mosquitoes may actually feed upon raptors at higher rates than expected in the case of random feeding on available birds.

Our study has limitations in that the number of bird species used was low, the experiment was conducted at a single location and blood meal analysis was not performed to rule out the possibility that individual mosquitoes had fed before entering the trap. Nevertheless, a limited number of such experiments is available in the literature and, to our knowledge, this is the first analysis of host feeding preferences comparing raptors with birds from other avian orders in Europe. Amongst the strengths of the experiment are the use of live-baited traps that, as in Balenghien et al. [22], allowed mosquitoes to feed on birds, thus providing additional information on engorgement rates and reducing the possibility of mosquito escapes [23]. In addition, traps were wide enough to allow movements like stomping, shaking or flapping which represent the natural behavior defenses of birds against mosquitoes [43]. Finally, different individuals of the same bird species were used to minimize the potential bias due to natural intra-specific odor variations [20]. We are thus confident that the conditions in which the present study was carried out were an acceptable representation of events occurring in natural contexts.

Multiple mechanisms, ranging from host defensive behavior to body temperature and size-related carbon dioxide emission, may be suggested to explain the group-related differences recorded in this study. Edman et al. [44] showed that host behavioral traits largely influenced the feeding success of *Culex nigripalpus* upon a range of avian species, whereby the highest engorgement rates were found in nocturnal raptors (Strigiformes) displaying weak mosquito dislodging behavior. Host defensive attitude can have important implications in the transmission of WNV, because birds tolerant to vectors may be more frequently exposed to infection. It is also well known that mosquitoes respond to heat sources, and that convection currents created by body heat affect the dispersal of semiochemicals, influencing mosquito attraction [20]. Birds exude heat from metabolic activity, resulting in body temperature increases of several degrees during the waking phase of a daily cycle. Interestingly, most species of owls show the reverse cycle, with the highest body temperatures at night, synchronous to peaks of mosquito activity [45]. Finally, carbon dioxide plays an important role in the activation and attraction for all hematophagous arthropods [20, 46], and several studies have shown that larger animals exhaling higher quantities of carbon dioxide positively correlates with mosquito attraction [19]. In our study, this has been clearly shown in the raptor group, whereby the peregrine falcon, the heaviest raptor bait, was the most attractive to mosquitoes.

The similar numbers of attracted mosquitoes in large and medium-sized non-raptor species was relatively surprising. We can hypothesize that mallards, the heaviest bait and the only waterfowl species in this study, were relatively unattractive to mosquitoes due to a combination of anatomical (i.e. the densely feathered apteria, an adaptation to insulate the body from cold water) [47] and behavioral traits (e.g. resting position), both limiting the amount of exposed skin and exhaled carbon dioxide. In general, wild ducks (Anseriformes) are deemed minor players in the circulation of WNV since experimental evidence has shown that they do not develop adequate viremia levels to infect most feeding mosquitoes [48, 49].

Other than *C. pipiens*, bird-baited traps attracted low numbers of other mosquito species with lower engorgement rates. This was expected since none of these species has a recognized feeding preference for birds (Table 1). Although WNV has been repeatedly isolated from *A. albopictus*, its vector role is questionable since only a small proportion of blood meals is actually obtained from avian hosts [50, 51]. In Italy, during national and regional surveillance activities from 2008 to 2012, none of the 1709 pools of *A. albopictus* analyzed were found to be positive for WNV [3].

The consequences of mosquito feeding patterns for the transmission of WNV to raptors are multiple and important. Different studies report that birds of prey are frequently infected by WNV during outbreaks in Europe and North America [7–11], suggesting that the contact rates between WNV vectors and raptors were not negligible. Furthermore, bird species of the order Strigiformes have been severely affected by Usutu virus (USUV), another member of the Japanese encephalitis serocomplex, which also has *C. pipiens* as the main vector in Europe [52]. Raptors are known to be highly susceptible to WNV, and some species belonging to the orders Falconiformes, Accipitriformes and Strigiformes showed high viremic titers during experimental infections, suggesting that they can be competent amplifying hosts, hence significant players in the transmission cycle of the virus [6, 53]. Finally, the appreciable feeding by *C. pipiens* on large raptor birds coupled with their susceptibility to WNV infection, raises interest in this group as a candidate target for official in vivo surveillance of WNV and other mosquito-borne flaviviruses, as a compliment to backyard poultry. In this perspective, sound collaboration between public health agencies and the mostly volunteer network of wildlife rehabilitation centers throughout Europe is advisable to make informative samples (e.g. obtained from long-term sedentary patients) sustainably available at a relatively low cost, thus reducing public investment in other labor-intensive actions [24, 54–57].

## Conclusion

In conclusion, our study shows that *C. pipiens* has a feeding preference for raptor birds, which concurs with the results of a recent seroepidemiological survey for WNV in a range of avian orders in northern Italy [24]. This is a step towards the better understanding of mosquito feeding patterns in natural bird communities in Europe. A larger number of similar studies including more repetitions, locations and bird species used as baits would be helpful to clarify the role of different avian taxa in the epidemiology of WNV and other zoonotic mosquito-borne flaviviruses.

## References

[1] Weissenböck H, Hubálek Z, Bakonyi T, Nowotny N (2010). Zoonotic mosquito-borne flaviviruses: Worldwide presence of agents with proven pathogenicity and potential candidates of future emerging diseases. Vet Microbiol.

[2] Rizzo C, Salcuni P, Nicoletti L, Ciufolini MG, Russo F, Masala R (2012). Epidemiological surveillance of west Nile neuroinvasive diseases in Italy, 2008 to 2011. Eurosurveillance.

[3] Engler O, Savini G, Papa A, Figuerola J, Groschup MH, Kampen H (2013). European surveillance for West Nile virus in mosquito populations. Int J Environ Res Public Health.

[4] Ozdenerol E, Taff GN, Akkus C (2013). Exploring the spatio-temporal dynamics of reservoir hosts, vectors, and human hosts of west Nile virus: A review of the recent literature. Int J Environ Res Public Health.

[5] Gray TJ, Webb CE (2014). A review of the epidemiological and clinical aspects of West Nile virus. Int J Gen Med.

[6] Pérez-Ramírez E, Llorente F, Jiménez-Clavero MÁ (2014). Experimental infections of wild birds with West Nile virus. Viruses.

[7] Fitzgerald SD, Patterson JS, Kiupel M, Simmons HA, Grimes SD, Sarver CF (2003). Clinical and pathologic features of West Nile virus infection in native North American owls (Family Strigidae). Avian Dis.

[8] Gancz AY, Campbell DG, Barker IK, Lindsay R, Hunter B (2004). Detecting West Nile virus in owls and raptors by an antigen-capture assay. Emerg Infect Dis.

[9] Wünschmann A, Shivers J, Bender J, Carroll L, Fuller S, Saggese M (2004). Pathologic findings in red-tailed hawks (*Buteo jamaicensis*) and Cooper’s hawks (*Accipiter cooper*) naturally infected with West Nile virus. Avian Dis.

[10] Busquets N, Bertran K, Costa TP, Rivas R, de la Fuente JG, Villalba R (2012). Experimental West Nile virus Infection in Gyr-Saker hybrid falcons. Vector Borne Zoonotic Dis.

[11] Wodak E, Richter S, Bagó Z, Revilla-Fernández S, Weissenböck H, Nowotny N (2011). Detection and molecular analysis of West Nile virus infections in birds of prey in the eastern part of Austria in 2008 and 2009. Vet Microbiol.

[12] Rizzoli A, Bolzoni L, Chadwick E a, Capelli G, Montarsi F, Grisenti M (2015). Understanding West Nile virus ecology in Europe: *Culex pipiens* host feeding preference in a hotspot of virus emergence. Parasit Vectors.

[13] Apperson CS, Hassan HK, Harrison BA, Savage HM, Aspen SE, Farajollahi A (2004). Host feeding patterns of established and potential mosquito vectors of West Nile virus in the eastern United States. Vector Borne Zoonotic Dis.

[14] Chaves LF, Harrington LC, Keogh CL, Nguyen AM, Kitron UD (2010). Blood feeding patterns of mosquitoes: random or structured?. Front Zool.

[15] Radrova J, Seblova V, Votypka J (2013). Feeding behavior and spatial distribution of *Culex* mosquitoes (Diptera: Culicidae) in wetland areas of the Czech Republic. J Med Entomol.

[16] Janousek WM, Marra PP, Kilpatrick A (2014). Avian roosting behavior influences vector-host interactions for West Nile virus hosts. Parasit Vectors.

[17] Bashar K, Tuno N, Ahmed T, Howlader A (2012). Blood-feeding patterns of *Anopheles* mosquitoes in a malaria-endemic area of Bangladesh. Parasit Vectors.

[18] Takken W (1999). Chemical signals affecting mosquito behaviour. Invertebr Reprod Dev.

[19] Burkett-Cadena ND, Bingham AM, Porterfield C, Unnasch TR (2014). Innate preference or opportunism: mosquitoes feeding on birds of prey at the Southeastern Raptor Center. J Vector Ecol.

[20] Takken W, Verhulst NO (2013). Host preferences of blood-feeding mosquitoes. Annu Rev Entomol.

[21] Smallegange RC, Takken W, Takken W, Knols BGJ (2010). Host-seeking behaviour of mosquitoes: responses to olfactory stimuli in the laboratory. Olfaction in vector-host interactions.

[22] Balenghien T, Fouque F, Sabatier P, Bicout DJ (2006). Horse-, bird-, and human-seeking behavior and seasonal abundance of mosquitoes in a West Nile virus focus of southern France. J Med Entomol.

[23] Service W, Service W, Chapman & Hall, London UK (1993). Sampling adults by animal bait catches and by animal-baited traps. Mosquito Ecology: Field Sampling methods.

[24] Victoriano I, Rossi L, Tomassone L, Grego E, Mosca A, Silvano F (2015). Serological investigation of Usutu and West nile viruses in wild and domestic birds in Northwestern Italy, 2012–2014. 2nd Conf.

[25] Komnenou A, Georgopoulou T, DA Savvas I (2005). A retrospective study of presentation, treatment and outcome of free-ranging raptors in Greece (1997–2000). J Zoo Wildl Med.

[26] Nemeth NM, Kratz GE, Bates R, Scherpelz JA, Bowen RA, Komar N (2009). Clinical evaluation and outcomes of naturally acquired West Nile virus infection in raptors. J Zoo Wildl Med.

[27] Molina-Lopez RA, Casal J, Darwich L (2014). Specie-specific outcomes of wild raptors attended at a Wildlife rehabilitation centre in Catalonia (1997–2005). Am J Anim Vet Sci.

[28] Severini F, Toma L, di Luca M, Romi R (2009). Identification of the adult stages of the Italian mosquitoes (Diptera, Culicidae). Fragm Entomol Università degli Studi di Roma La Sapienza.

[29] Stojanovich CJ, Scott HG. Mosquitoes of Italy: mosquitoes of the Italian biogeographic area which includes the Republic of Malta, the French Island of Corsica and all of Italy except the Far-Northern Provinces. Stojanovich CJ, editor. 1997.

[30] Pinheiro J, Bates D (2000). Mixed-Effects Models in S and S-PLUS.

[31] Zuur A, Leno EN, Walker N, Saveliev AA, Smith GM (2009). Mixed effects models and extensions in ecology with R.

[32] Burnham KP, Anderson D (2002). Model Selection and Multimodel Inference: A Practical Information-Theoretic Approach.

[33] Nakagawa S, Schielzeth H (2013). A general and simple method for obtaining R 2 from generalized linear mixed-effects models. Methods Ecol Evol..

[34] Pinheiro J, Douglas B, Debroy S, Sarkan D (2015). Linear and nonlinear mixed effects models. R package version 3.1–120.

[35] Bates D, Maechler M, Bolker B, Walker S (2015). Fitting linear mixed-effects models using lme4. J Stat Softw.

[36] Barton K (2014). MuMln: Multi-model inference. R package version 1.10.5.

[37] R Core Team (2015). R: A language and environment for statistical computing.

[38] Farajollahi A, Fonseca DM, Kramer LD, Marm Kilpatrick A (2011). “Bird biting” mosquitoes and human disease: a review of the role of *Culex pipiens* complex mosquitoes in epidemiology. Infect Genet Evol.

[39] Savage HM, Aggarwal D, Apperson CS, Katholi CR, Gordon E, Hassan HK (2007). Host choice and West Nile virus infection rates in blood-fed mosquitoes, including members of the Cu*lex pipiens* complex, from Memphis and Shelby County, Tennessee, 2002–2003. Vector Borne Zoonotic Dis.

[40] Hamer GL, Kitron UD, Goldberg TL, Brawn JD, Loss SR, Ruiz MO, Hayes DB, Walker E (2009). Host selection by *Culex pipiens* mosquitoes and west nile virus amplification. Am J Trop Med Hyg.

[41] Estep LK, McClure CJW, Burkett-Cadena ND, Hassan HK, Hicks TL, Unnasch TR, Hill G (2011). A multi-year study of mosquito feeding patterns on avian hosts in a southeastern focus of Eastern equine encephalitis virus. Am J Trop Med Hyg.

[42] Thiemann TC, Lemenager DA, Kluh S, Carroll BD, Lothrop HD, Reisen WK (2012). Spatial variation in host feeding patterns of *Culex tarsalis* and the *Culex pipiens* complex (Diptera: Culicidae) in California. J Med Entomol.

[43] Darbro JM, Harrington LC (2007). Avian defensive behavior and blood-feeding success of the West Nile vector mosquito, *Culex pipiens*. Behav Ecol.

[44] Edman JD, Webber LA, Schmid A (1974). Effect of host defenses on feeding pattern of mosquitoes. J Parasitol.

[45] Collins C (1991). Diurnal body temperature cycle in the northern hawk-owl (*Surnia ulula*). J Raptor Res.

[46] Torr SJ, Mangwiro TNC, Hall DR (2006). The effects of host physiology on the attraction of tsetse (Diptera: Glossinidae) and *Stomoxys* (Diptera: Muscidae) to cattle. Bull Entomol Res.

[47] Proctor NS, Lynch PJ. Manual of Ornithology: Avian Structure & Function. Yale University Press; 1998. ISBN 0300076193, 9780300076196.

[48] Komar N, Langevin S, Hinten S, Nemeth N, Edwards E, Hettler D, Davis B, Bowen R, Bunning M (2003). Experimental infection of North American birds with the New York 1999 strain of West Nile virus. Emerg Infect Dis.

[49] Hayes EB, Komar N, Nasci RS, Montgomery SP, O’Leary DR, Campbell G (2005). Epidemiology and transmission dynamics of West Nile Virus disease. Emerg Infect Dis.

[50] Faraji A, Egizi A, Fonseca DM, Unlu I, Crepeau T, Healy SP (2014). Comparative host feeding patterns of the Asian tiger mosquito, *Aedes albopictus*, in urban and suburban northeastern USA and implications for disease transmission. PLoS Negl Trop Dis.

[51] Richards SL, Ponnusamy L, Unnasch TR, Hassan HK, Apperson C (2006). Host-feeding patterns of *Aedes albopictus* (Diptera: Culicidae) in relation to availability of human and domestic animals in suburban landscapes of central North Carolina. J Med Entomol.

[52] Steinmetz HW, Bakonyi T, Weissenböck H, Hatt JM, Eulenberger U, Robert N (2011). Emergence and establishment of Usutu virus infection in wild and captive avian species in and around Zurich, Switzerland-Genomic and pathologic comparison to other central European outbreaks. Vet Microbiol.

[53] Ziegler U, Angenvoort J, Fischer D, Fast C, Eiden M, Rodriguez AV (2013). Pathogenesis of West Nile virus lineage 1 and 2 in experimentally infected large falcons. Vet Microbiol.

[54] Victoriano Llopis I, Rossi L, Di Gennaro A, Mosca A, Teodori L, Tomassone L (2015). Further circulation of West Nile and Usutu viruses in wild birds in Italy. Infect Genet Evol.

[55] Nemeth N, Kratz G, Edwards E, Scherpelz J, Bowen R, Komar N (2007). Surveillance for West Nile Virus in Raptors, Colorado. Emerg Infect Dis.

[56] Nemeth NM, Beckett S, Edwards E, Klenk KN (2007). Avian mortality surveillance for West Nile virus in Colorado. Am J Trop Med Hyg.

[57] Manarolla G, Bakonyi T, Gallazzi D, Crosta L, Weissenböck H, Dorrestein GM (2010). Usutu virus in wild birds in northern Italy. Vet Microbiol.

[58] Gomes B, Sousa CA, Vicente JL, Pinho L, Calderón I, Arez E (2013). Feeding patterns of molestus and pipiens forms of *Culex pipiens* (Diptera: Culicidae) in a region of high hybridization. Parasit Vectors.

[59] Valerio L, Marini F, Bongiorno G, Facchinelli L, Pombi M, Caputo B (2008). Blood-feeding preferences of *Aedes albopictus* (Diptera: Culicidae) in urban and rural settings within the province of Rome, Italy. Parassitologia.

[60] Greenberg J, Lujan D, DiMenna M, Wearing HJ, Hofkin BV (2013). Identification of blood meal sources in *Aedes vexans* and *Culex quinquefasciatus* in Bernalillo County, New Mexico. J Insect Sci.

[61] Burkett-Cadena ND, Graham SP, Hassan HK, Guyer C, Eubanks MD, Katholi CR (2008). Blood feeding patterns of potential arbovirus vectors of the genus *Culex* targeting ectothermic hosts. Am J Trop Med Hyg.

[62] Medlock JM, Snow KR, Leach S (2005). Potential transmission of West Nile virus in the British Isles: An ecological review of candidate mosquito bridge vectors. Med Vet Entomol.

